# Using rubber stamps and mobile phones to help understand and change antibiotic prescribing behaviour in private sector primary healthcare clinics in Kenya

**DOI:** 10.1136/bmjgh-2019-001422

**Published:** 2019-09-29

**Authors:** Bernadette Kleczka, Pratap Kumar, Mercy Karimi Njeru, Anita Musiega, Phoebe Wekesa, Grace Rabut, Michael Marx

**Affiliations:** 1 Haematology and Blood Transfusion, Muhimbili University of Health and Allied Sciences, Dar es Salaam, United Republic of Tanzania; 2 Heidelberg Institute of Global Health, UniversitatsKlinikum Heidelberg, Heidelberg, Germany; 3 Health-E-Net Limited, Nairobi, Kenya; 4 Institute of Healthcare Management, Strathmore University Business School, Nairobi, Kenya; 5 Centre for Public Health Research, Kenya Medical Research Institute, Nairobi, Kenya; 6 Division of HIV, TB and Malaria, Ministry of Health and Sanitation, Kitui, Kenya

**Keywords:** infections, diseases, disorders, injuries, health systems, public health, health services research

## Abstract

**Background:**

Antibiotic use in primary care can drive antimicrobial resistance (AMR) in the community. However, our understanding of antibiotic prescribing in low- and middle-income countries (LMICs) stems mostly from hospital-based studies or prescription/sales records, with little information available on routine primary care practices. We used an innovative, paper-to-digital documentation approach to deliver routine data and understand antibiotic use for common infections in low-resource primary healthcare clinics (PHCs).

**Methods:**

Rubber stamps were introduced in nine private sector PHCs serving Nairobi’s informal settlements to ‘print-on-demand’ clinical documentation templates into paper charts. The intervention included one mobile phone per PHC to take and share images of filled templates, guideline compilation booklets and monthly continuing medical education (CME) sessions. Templates for upper respiratory tract (URTI), urinary tract (UTI), sexually transmitted (STI) and gastrointestinal infection (GI) management were used in eight PHCs. Information in templates from 889 patient encounters was digitised from smartphone images, analysed, and fed back to clinicians during monthly CME sessions. UTI charts (n=130 and 96, respectively) were audited preintervention and postintervention for quality of clinical documentation and management.

**Results:**

Antibiotics were prescribed in 94.3%±1.6% of all patient encounters (97.3% in URTI, 94.2% in UTI, 91.6% in STI and 91.3% in GI), with 1.4±0.4 antibiotics prescribed per encounter. Clinicians considered antibiotic use appropriate in only 58.6% of URTI and 47.2% of GI cases. While feedback did not affect the number of antibiotics prescribed for UTIs, the use of nitrofurantoin, an appropriate, narrow-spectrum antibiotic, increased (9.2% to 29.9%; p<0.0001) and use of broad spectrum quinolones decreased (30.0% to 16.1%; p<0.05).

**Conclusion:**

Antibiotic use for common infections is high in private sector PHCs in Kenya, with both knowledge and ‘know-do’ gaps contributing to inappropriate prescription. Paper-based templates in combination with smartphone technologies can sustainably deliver routine primary care case management data to support the battle against AMR.

**Key questions**

**What is already known?**

- Antimicrobial resistance is a global problem with a particularly heavy burden expected to be borne by low- and middle-income countries (LMICs).
- Increased consumption of antibiotics in primary care can increase resistance at individual, community and national levels, but few data on antibiotic use in primary care are available from LMICs.

**What are the new findings?**

- Antibiotic prescription for commonly encountered infectious conditions is very common in low-resource primary care settings in Kenya, with more than one antibiotic prescribed on average per patient encounter.
- Gaps in clinical knowledge as well as ‘know-do’ gaps contribute to inappropriate use of antibiotics by non-physician clinicians.
- Innovative and sustainable approaches to collect routine digital data on clinical case management, combined with proven audit and feedback cycles, can improve adherence to clinical practice guidelines, including rational use of antibiotics.

**What do the new findings imply?**

- Antimicrobial stewardship efforts should focus on both improving clinician knowledge on relevant clinical practice guidelines and care pathways, as well as promoting strategies to improve clinician adherence to their recommendations.
- Routine data on primary care practices can be a powerful tool to both monitor use of antibiotics and address gaps through regular audit and feedback cycles or clinical decision support systems.

## Introduction

The impact of emerging global antimicrobial resistance (AMR) is likely to be particularly large in low-income and middle-income countries (LMICs).^1 2^ Overuse of antibiotics in primary healthcare is clearly linked to the development and sustenance of resistance mechanisms in bacteriae.^3^ While access to second-line and third-line antibiotics is still limited in LMICs, antibiotic overuse is believed to be a problem, particularly in the overcrowded areas of urban informal settlements of fast-growing cities.^4^ As antibiotic overuse continues to rise in emerging economies,^5^ it is recognised that development of new antibiotics alone is unlikely to lead to a sustainable solution, and greater emphasis needs to be placed on rational use in human health and other sectors.^6^

Most of our knowledge on antibiotic use in LMICs stems from hospital-based studies or prescription/sales records.^7–9^ Little is known about antibiotic use in primary care settings in LMICs, and few studies have explored reasons for inappropriate antibiotic prescription by primary care clinicians.^7^ Reasons for antibiotic overuse by clinicians in LMICs can fall under either ‘knowledge gaps’, such as lack of awareness or unfamiliarity with current clinical practice guidelines or ‘know-do gaps’, where provider practices diverge from what they know they should do.^10^ Know-do gaps may result from barriers of attitude (eg, inertia of previous practice, lack of motivation, inadequate leadership) or behaviour (eg, patient preferences, lack of time or resources like appropriate drugs and diagnostics).^11^ Distinguishing between knowledge and know-do gaps, and understanding their causes, is critical for developing effective strategies to counter AMR.

Provider training and/or clinical decision support (CDS) systems could close knowledge gaps,^12^ but overcoming barriers of attitude or behaviour may require investment in quality improvement (QI) efforts through routine audit and feedback strategies.^13 14^ Routine data on the management of common infectious diseases can provide rich insights into the problem of antibiotic overuse in primary care settings, but in LMIC settings such data are difficult to collect,^15^ and efforts to link routine data to regular audit and feedback cycles are rare.^13^ Furthermore, the private sector is playing an increasingly vital role in healthcare delivery in LMICs but with little support for systematic QI across a fragmented healthcare market.^16 17^ Calls for cross-sector solutions to combat AMR are just beginning,^2^ but the collection and use of routine data on infectious disease management are necessary across different contexts of primary healthcare delivery in LMIC settings.

While computer-based electronic medical record (EMR) systems are used to provide routine data on clinical care in high-income countries, paper is still a commonly used interface for documentation in LMICs.^18^ Besides the challenges of implementing EMR systems in low-resource settings,^19^ little is known about the quality of data entered into electronic systems in LMIC contexts.^20^ The recent revolution in access to mobile technologies provides a great opportunity to reach and support frontline health workers in LMICs.^21^ A combination of mobile technologies and paper-based documentation could potentially overcome the limitations of using traditional EMR systems in providing routine clinical management data.^22^

The Guidelines Adherence in Slums Project used such a ‘paper-to-digital’ approach to generate routine data on patient care delivered in low-resource primary care settings.^23^ Rubber stamps were used to print templates of clinical case management into paper charts, providing non-physician clinicians in private sector primary healthcare clinics (PHCs) with a standard, evidence-based checklist and documentation tool that could be used during consultations. While templates are designed for automatic extraction of data entered in paper using computer vision algorithms on low-end smartphones, the use of templates in and of itself improved quality of clinical documentation for three non-communicable diseases.^23^ This publication reports on the use of templates to support and deliver routine data on the management of commonly encountered infectious diseases in PHCs.

## Methods

The Guidelines Adherence in Slums Project aimed to test the usability, acceptability and effectiveness of paper-based clinical documentation tools in improving quality of outpatient care in private sector PHCs serving the urban informal settlements of Nairobi, Kenya.^23^ While usability and acceptability were assessed using qualitative tools (manuscript in preparation), this paper focuses on effectiveness of the intervention in improving quality of clinical documentation and care. Ten facilities were purposively targeted through existing links or partnerships, and two exclusion criteria were used: high staff turnover (one facility excluded) and any ongoing QI programmes targeting clinical quality for general outpatient conditions (none excluded). The management at each PHC was free to select clinical conditions to be covered in the project from five infectious diseases (upper respiratory tract infections (URTI), urinary tract infections (UTI), sexually transmitted infections (STI) and gastrointestinal infections (GI) and malaria), three non-communicable diseases (hypertension, diabetes and chronic respiratory disease), pregnancy-related conditions (UTI, STI and hypertension) and referral (maternal and paediatric cases). This study focuses on data from URTI, UTI, STI and GI cases.

**Figure 1:**
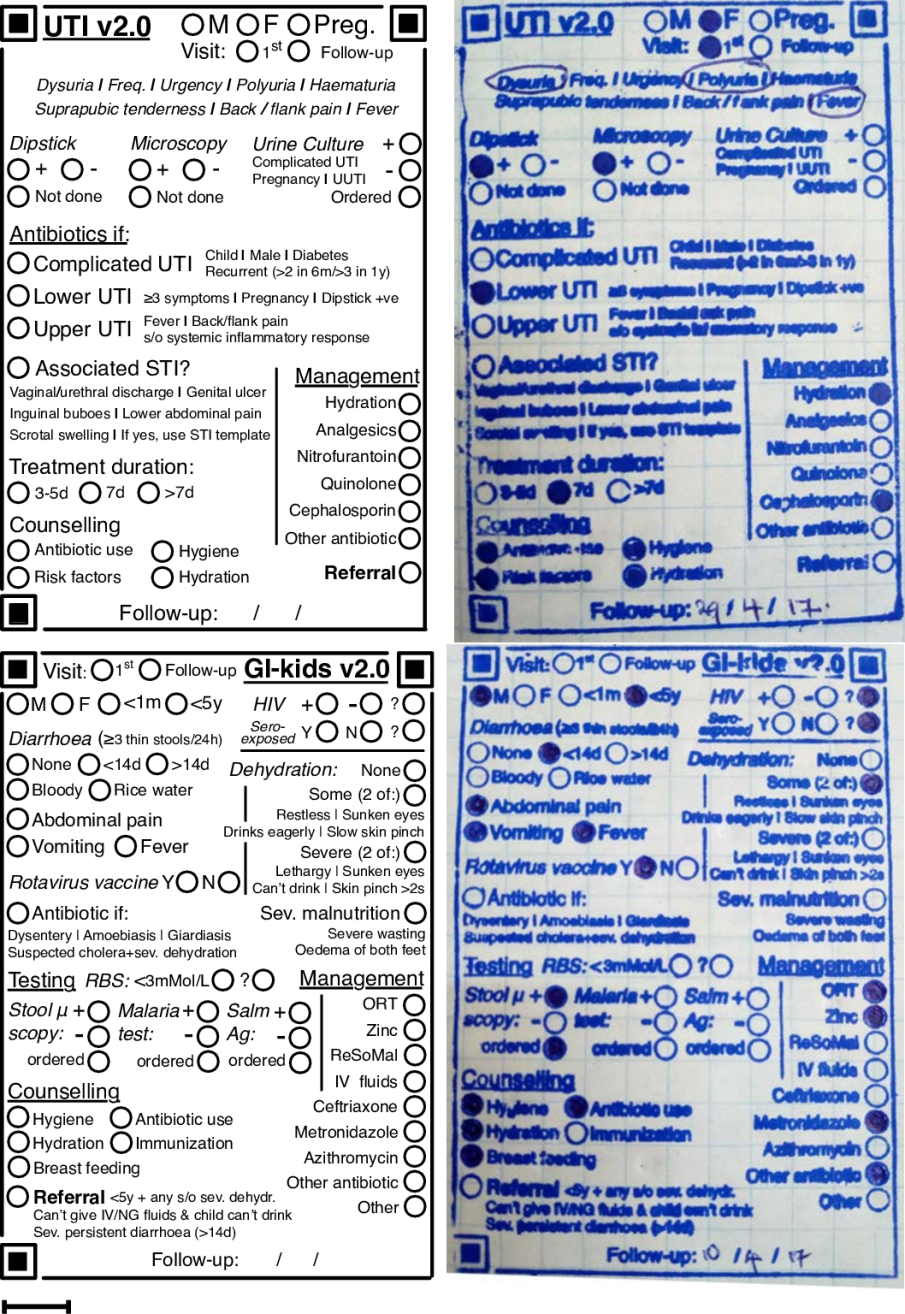
Templates for UTI and GI (left) and their corresponding rubber stamp versions in paper charts (right). Scale bar: 1 cm. GI, gastrointestinal infection; UTI, urinary tract infection.

The intervention included four elements: Rubber stamp templates for documenting management of selected conditions (figure 1), compilations of the relevant clinical practice guidelines, one low-budget (∼$70) Android smartphone to each facility and one continuing medical education (CME) session at each facility every month. Guideline compilation booklets were requested by the clinicians/managers of the PHCs with the intention of referring to them as needed; their use was not measured. Smartphone images of templates were taken by clinical or non-clinical staff (depending on the workflows in each PHC) and automatically synched to a cloud server. While a smartphone app for automatic data extraction has since been deployed, all template data in this study were manually extracted from images. Each clinic received at least six CME sessions, with occasional interruptions due to security concerns related to the 2017 elections in Kenya. CME sessions included a review of specific guideline topics related to the templates (selected by the PHC) and monthly data reports on the management of different conditions (eg, number of cases seen, prescription rates, antibiotics used and so on).

Besides routine collection and analysis of template data, paper-based charts were compared before and after the intervention. A clinical audit of UTI cases was done in PHCs where charts were maintained in the facility. Audits were done manually by one member of the research team (PW) and reviewed by another member (BK). Ministry of Health reporting registers were used to identify visits for UTIs. UTI in non-pregnant women (<65 y), pregnant women and men were included for analysis. UTI in patients with diabetes, and visits where the diagnosis of UTI was not clearly documented were excluded. The target was 96 charts each from the pre-intervention and post-intervention periods to detect a 20% difference in documentation scores with 95% confidence and 80% power. Pre-intervention data for PHC #2 spanned one year, while data for PHCs #5 and #6 were available for nine months. Postintervention audits began one month after implementation of the intervention. Post-intervention UTI cases were audited over 11 months in PHC #2 (stopped before elections in Kenya) and over seven months in PHCs #5 and #6 (before dropout to implement an EMR system).

### Analysis of clinical documentation

A scoring system was developed to compare documentation in charts and templates across four dimensions—general data, assessment, testing and management (table 1). Control criteria included information not possible to document in templates (eg, narrative information like presenting complaints, continuous data like vital signs). One point was awarded for information available in the chart for each criterion. Scoring was initially done in Microsoft Excel, and data exported to R^24^ for analysis. Variance in the data was measured using f-tests. Data are reported as weighted means and SD; t-tests were used to compare means, and χ² tests to compare proportions.

**Table 1:** Scoring criteria for clinical documentation

Dimension	Information	Chart	Template
Scoring dimensions for information in UTI charts and templates			
General data	Sex (non-pregnant patients only)	Documented on first page	Circle filled
	Visit information (First/Follow-up/ANC visit)	Documented in encounter	Circle filled
	Pregnancy details—trimester	Documented in encounter	Circle filled
	Pregnancy details—gravidity, Parity	Documented in encounter	Circle filled
Assessment	UTI type (lower/upper/complicated UTI)	Clear documentation of subdiagnosis	Any subdiagnosis filled
	Possible associated STI	Documentation of STI symptoms (positive or negative)	Circle filled
Testing	Urine dipstick/microscopy	Test mentioned in encounter	Circle filled
	Urine culture	Test mentioned in encounter	Circle filled
Management	Prescription/Management instructions	Clear management orders in encounter	Any management circles filled
	Hydration instructions	Noted in encounter as management or counselling	Circle filled in either management or counselling
	Treatment duration	Treatment duration clearly documented	Circle filled
	Counselling	Any counselling (eg, antibiotic use, risk factors, hygiene) documented	Any counselling circles filled
# Criteria	10 (11 in pregnant women)		
Control dimensions (information documented outside templates)			
General data	Presenting complaints	Noted in encounter	N/A
Vital signs	Blood pressure	Exact values documented	N/A
	Heart rate		N/A
	Temperature		N/A
	Weight		N/A
Follow-up	Follow-up instructions	Follow-up date documented	N/A
# Criteria	6		

ANC, antenatal care; STI, sexually transmitted infection; UTI, urinary tract infection.

### Analysis of adherence to clinical practice guidelines

Adherence to guidelines was determined using two measures: appropriateness of diagnosis and appropriateness of antibiotic use. Criteria for both were developed from Kenyan and international guidelines for diagnosis and management of UTIs. All charts were assessed for documentation of presence of dysuria and frequency (likelihood of UTI >90%), presence of urethral/vaginal discharge (UTI less likely) and testing.^25^ Criteria for diagnosis of UTI in non-pregnant women were: documentation of three or more UTI symptoms or two symptoms and a positive test (microscopy or dipstick).^25^ In pregnant women, the criterion was a positive dipstick only, because of the risk of acute pyelonephritis in late pregnancy in mothers with asymptomatic bacteriuria.^26^ In men, the criteria were dysuria and positive urine dipstick.^27^

Due to limited data on AMR in the settings of this study,^28^ we used the following criteria for appropriateness of antibiotic prescription: (1) use of narrow-spectrum antibiotics for lower UTIs. Nitrofurantoin is recommended as first-line therapy for lower UTI in non-pregnant women; fosfomycin was not available at the time of the study.^25 29^ (2) Ampicillin is not recommended in non-pregnant women with lower UTI due to global and regional resistance rates.^1^ (3) In pregnancy, nitrofurantoin is recommended as first-line treatment except in the third trimester,^25^ when amoxicillin, erythromycin^29^ or third generation cephalosporin are recommended.^26^ (4) In men, ciprofloxacin is recommended as first-line antibiotic, with nitrofurantoin as an alternative.^27^ Specific guidelines and literature were used to classify antibiotics as broad-spectrum.^25 30^

### Ethical approval

Ethical approval for the study was obtained from the ethical review committee of Strathmore University and the ethics committee of Heidelberg University. Template data did not contain any patient identifiers, and patient names in charts were redacted before data collection for audits. The manuscript uses the SQUIRE 2.0 standards for reporting.^31^

### Patient and public involvement

There were no patients involved in the study. Study participants included clinicians and managers of private sector PHCs in urban slums, who were involved in the development of research questions and tools. They were also the recipients of reports of facility-level data and CME sessions.

## Results

Out of 10 facilities targeted, one was excluded due to high staff turnover. All other nine were provided with rubber stamp templates to document commonly encountered conditions. One facility focused only on the three non-communicable diseases. Templates for the four infectious diseases which are the subject of this study (URTI, UTI, STI, GI) were introduced in eight PHCs. Of these, one did not provide any data on use and one closed during the study. Template data from a PHC were included for analysis if templates were used to manage at least 10 encounters per condition per clinic (one excluded). Data from 889 templates were digitised and analysed across the five facilities using templates for managing infectious diseases. This included 261 cases of URTI (across three clinics), 360 cases of UTI (five clinics), 107 STI cases (three clinics) and 161 GI cases (three clinics).

### Antibiotic prescription rates

Antibiotics were prescribed in 94.3%±1.6% of the 889 patient encounters documented with templates, across the four infectious diseases and five PHCs (table 2). Antibiotic prescription rates did not vary greatly between facilities. Antibiotics were prescribed in 97.3%±2.3% of all URTI encounters, 94.2%±3.8% of UTI encounters, 91.6%±1.1% of STI encounters and 91.3%±1.4% of GI encounters. Templates contained a list of antibiotic classes that were appropriate for the condition, with corresponding bubbles that were to be shaded if an antibiotic of that class was prescribed (figure 1). Actual prescription data were not collected, and it was assumed that only one antibiotic from each class was prescribed. Approximately one antibiotic was prescribed per encounter for URTI and UTI (1.17±0.05 and 1.14±0.11, respectively). STI case management involved 2.55±0.31 antibiotic or antifungal agents (not including topical antifungal creams) per encounter and GI case management 1.40±0.22 antibiotics per encounter.

**Table 2:** Antibiotic use for common outpatient infectious diseases documented using rubber stamp templates

		n	Overall use of a'biotics (%)	# a'biotics/ encounter	Penicillin G Benzathine (%)	Amoxicillin (%)	Amoxiclav (%)	Cephalosporin (%)	Azi-/ Erythromycin (%)	Cipro-/ Norfloxacin (%)	Nitrofurantoin (%)	Metronidazole (%)	Other a'biotic (%)
URTI	PHC#1	131	99.2	1.2	2.3	20.8	49.2	27.7	5.4	n.a.	n.a.	n.a.	17.7
	PHC#2	35	97.1	1.2	0.0	23.5	17.6	52.9	5.9	n.a.	n.a.	n.a.	20.6
	PHC#3	95	94.7	1.1	8.9	51.1	23.3	21.1	4.4	n.a.	n.a.	n.a.	6.7
	All clinics	261	97.3	1.2	4.3	31.9	35.8	28.7	5.1	n.a.	n.a.	n.a.	14.2
UTI	PHC#1	59	94.9	1.1	n.a.	8.9	n.a.	30.4	0.0	26.8	19.6	n.a.	28.6
	PHC#2	71	94.4	1.4	n.a.	22.4	n.a.	38.8	0.0	11.9	34.3	n.a.	40.3
	PHC#3	123	95.9	1.1	n.a.	1.7	n.a.	19.5	11.9	21.2	53.4	n.a.	11.0
	PHC#4	84	95.2	1.1	n.a.	11.3	n.a.	31.3	3.8	10.0	37.5	n.a.	17.5
	PHC#5	23	78.3	0.8	n.a.	n.a.	n.a.	11.1	n.a.	33.3	44.4	n.a.	11.1
	All clinics	360	94.2	1.1	n.a.	9.1	n.a.	27.4	5.0	18.3	39.8	n.a.	21.2
STI	PHC#1	10	90.0	2.2	0.0	0.0	n.a.	0.0	33.3	n.a.	n.a.	33.3	11.1
	PHC#3	78	92.3	2.5	36.1	0.0	n.a.	0.0	61.1	15.3	n.a.	36.1	23.6
	PHC#4	19	89.5	3.1	17.6	11.8	n.a.	0.0	64.7	0.0	n.a.	58.8	0.0
	All clinics	107	91.6	2.6	29.6	2.0	n.a.	0.0	59.2	11.2	n.a.	39.8	18.4
GI	PHC#1	30	93.3	1.8	n.a.	n.a.	n.a.	50.0	7.1	n.a.	n.a.	96.4	42.9
	PHC#2	47	89.4	1.3	n.a.	n.a.	n.a.	11.9	0.0	n.a.	n.a.	52.4	76.2
	PHC#3	84	91.7	1.3	n.a.	n.a.	n.a.	42.9	26.0	n.a.	n.a.	59.7	7.8
	All clinics	161	91.3	1.4	n.a.	n.a.	n.a.	35.4	15.0	n.a.	n.a.	64.6	34.0

PHC, primary healthcare clinic.

### ‘Ideal’ versus ‘actual’ antibiotic use

Templates for URTI and GI (figure 1) included a checklist of criteria for antibiotic use, and a corresponding bubble to shade if the clinician thought antibiotic use was justified in the case. This ‘Antibiotics if’ bubble was shaded in only 58.6% of URTI and 47.2% of GI cases where antibiotics were prescribed. The bubble was shaded in only three cases where antibiotics were not used (two for URTI and one for GI).

### Completeness of documentation

**Figure 2:**
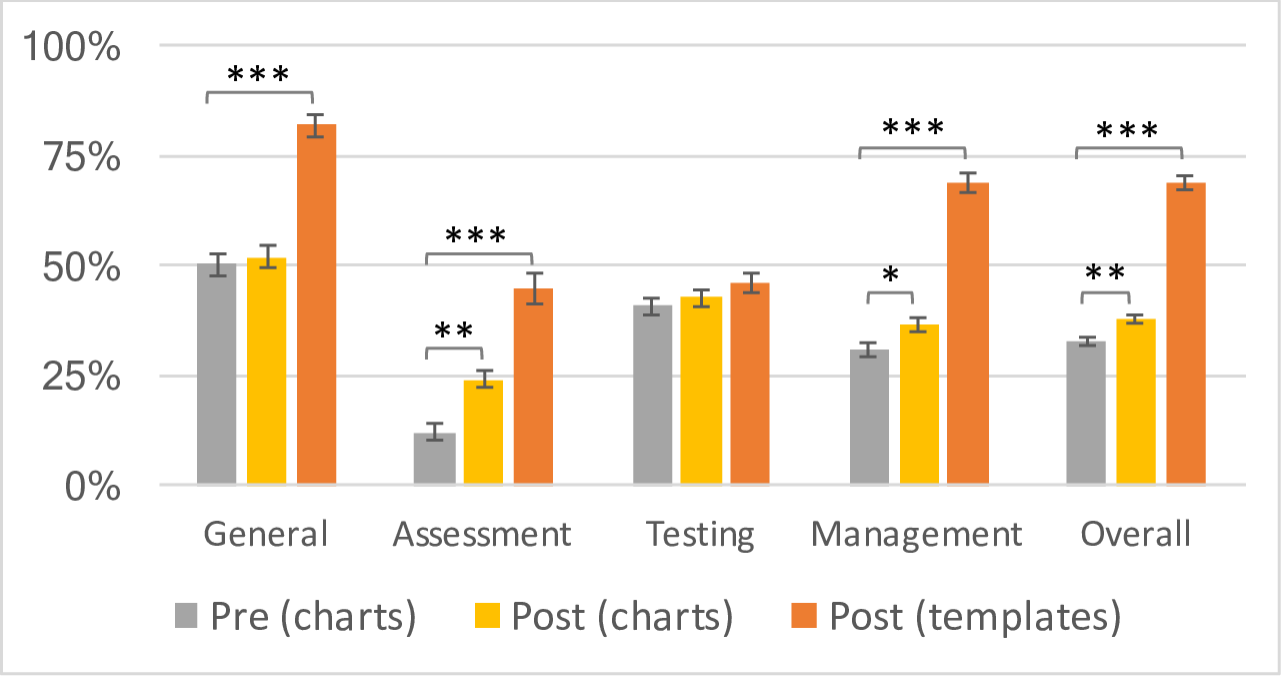
UTI documentation scores in charts (preintervention in grey and postintervention in yellow) and templates (orange). UTI, urinary tract infection.

Completeness of clinical documentation was assessed through a pre- and post-audit of UTI charts, using criteria presented in table 1. Charts were available for audit from three of the six PHCs (#2, #5 and #6) using rubber stamp templates for managing UTI. The other three PHCs used patient-held notebooks for documentation, which were not available to audit. Total 130 charts of UTI in the pre-intervention period and 96 in post-intervention period were audited (figure 2). Templates were used in 87 of 96 post-intervention charts audited, a usage rate of 90.6%.

Completeness of chart documentation improved slightly but significantly from 33.0%±1.0% preintervention to 38.3%±1.4% postintervention (t=−3.09, p<0.01). The greatest improvements in documentation scores were seen when templates were used. Overall template documentation scores (69.5%±1.7%) were significantly higher than both preintervention charts (t=−19.61, p<0.00001) and postintervention charts (t=−14.28, p<0.00001). Template scores were significantly higher than postintervention charts across all dimensions of documentation—general patient information (81.8%±2.8% vs 52.3±2.8%; t=−7.48, p<0.00001), patient assessment (44.8%±2.0% vs 24.0±3.2%; t=−5.36, p<0.00001) and management (69.0%±2.6% vs 36.5±2.2%; t=−9.71, p<0.00001)—except testing (46.0%±2.1% vs 42.7.0±2.0%; t=−1.16, p>0.05). Non-template information (presenting complaints, vital signs, follow-up instructions) remained high in both preintervention and postintervention charts (97.7% vs 96.9%, respectively; t=0.38, p>0.05) suggesting that narrative documentation was not compromised by template use (data not shown).

### Appropriateness of diagnosis and management

Vaginal or urethral discharge, which reduce the likelihood of UTI,^25^ were documented in a significant number of patients diagnosed with UTI (29.2% preintervention and 33.3% postintervention). This was observed in all three PHCs where UTI charts were audited. The confounding of UTI and STI extended into management. Antibiotics for STI (eg, doxycycline, metronidazole, gentamycin) were prescribed in 57% and 55% of all UTI cases preintervention and postintervention, respectively. However, use of antibiotics for STI was accompanied by documentation of any STI symptom in only 45% and 55% of preintervention and postintervention UTI cases, respectively (χ^2^(1, n=192)=0.41, p>0.05).

**Figure 3:**
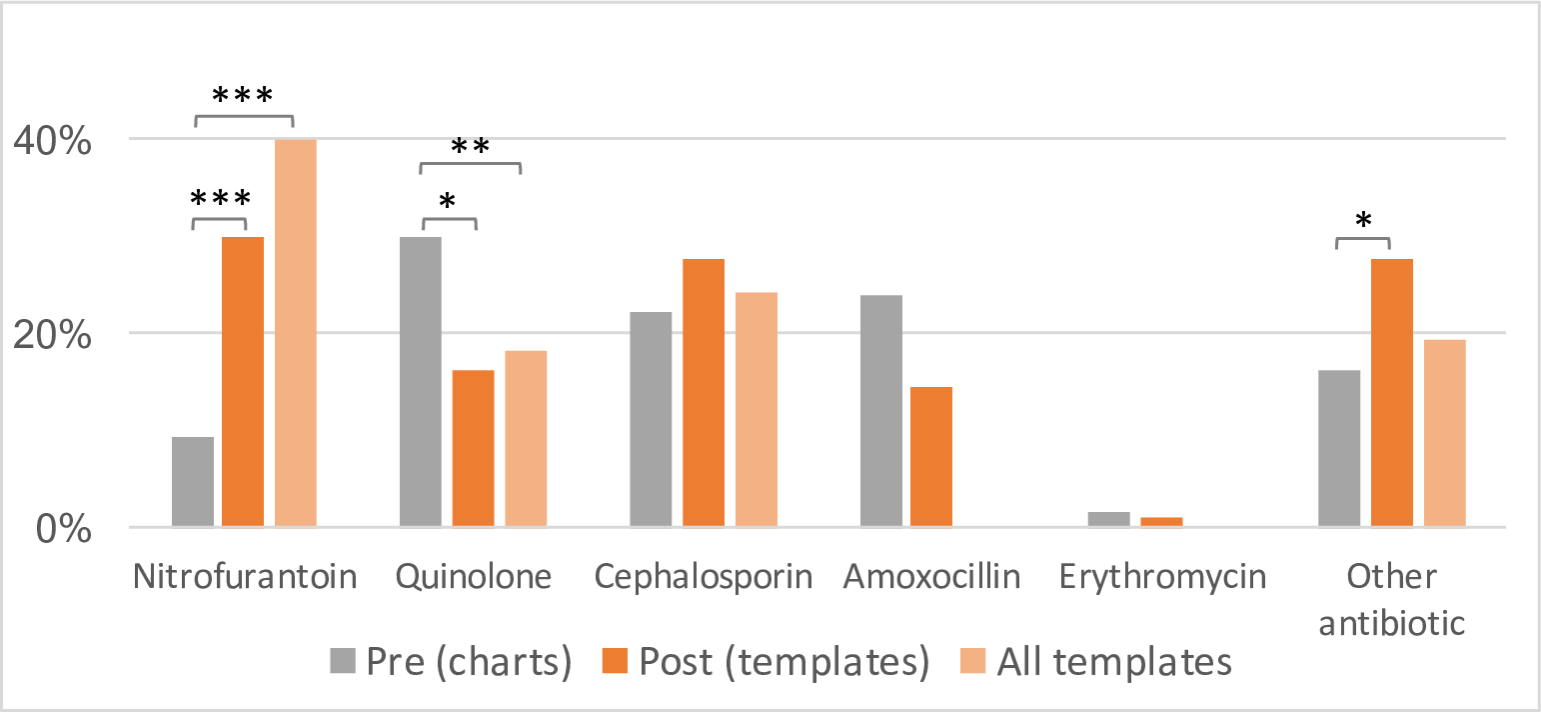
Antibiotic use in UTIs in preintervention charts (grey; n=130) and postintervention templates. Postintervention data are presented both from templates used in three facilities where charts were available for audit (orange; n=87) and across all facilities using templates (light orange; n=360). Amoxicillin and erythromycin were only included as options in templates for managing UTI in pregnancy; data reported here are from postintervention charts (n=96). UTI, urinary tract infection.

Chart audits revealed 1.75±0.08 antibiotics prescribed per UTI encounter in the preintervention period and 1.79±0.09 in the postintervention period (t=−0.32, p>0.05). These included both antibiotics indicated for UTIs such as nitrofurantoin, nalidixic acid or quinolones (0.97±0.05 vs 1.01±0.04) and antibiotic or antifungal agents typically prescribed for STIs such as metronidazole or fluconazole (0.78±0.07 vs 0.78±0.09). While the number of antibiotics prescribed per UTI encounter did not change postintervention, the type of antibiotics prescribed did change (figure 3).

The use of nitrofurantoin, a first line, narrow-spectrum antibiotic, increased from 9.2% of all preintervention encounters to 29.9% of all postintervention encounters using templates (χ^2^(1, n=217)=15.39, p<0.0001) in the three PHCs with audited chart data. Across all five PHCs using templates (n=360) nitrofurantoin was used in 39.8% of all UTI encounters where an antibiotic was prescribed. Use of quinolone antibiotics (ciprofloxacin or norfloxacin), only recommended for first-line use in men,^27^ reduced from 30.0% to 16.1% (χ^2^(1, n=217)=5.46, p=0.02); across all five PHCs using templates, quinolone use was documented in 18.3% of all UTI encounters. The use of amoxicillin or erythromycin, only recommended for first-line use in the third trimester of pregnancy, did not change significantly. Concordance of prescription data in charts and templates was tested in 33 cases. The overall concordance rate was 85.4%, with the lowest for quinolones (63.3%), possibly due to the lack of familiarity of non-physician clinicians with drug class, that is, quinolones, compared with drug names, for example, ciprofloxacin (unpublished qualitative data).

A subgroup analysis (data not shown) was conducted for UTIs in non-pregnant women (n=73 preintervention and 169 across all five PHCs postintervention), pregnant women (n=24 and 127) and men (n=33 and 43). Nitrofurantoin use increased significantly in both non-pregnant (9.6% to 29.6%; χ^2^(1, n=242)=11.32, p<0.001) and pregnant women (12.5% to 59.8%; χ^2^(1, n=151)=18.14, p<0.0001); no significant change was seen in UTI in men. Amoxicillin use in pregnant women decreased from 58.3% to 28.2% (χ^2^(1, n=140)=9.11, p<0.01); it was rarely prescribed in men (two cases preintervention and none post). No significant change was detectable in the prescription of quinolones or cephalosporins in any subgroup. Erythromycin was rarely used, either preintervention or postintervention, at any of the three facilities where charts were audited.

## Discussion

The few attempts to measure antibiotic use in primary care in LMICs have relied on reviews of prescription and sales records, simulated clients or observed encounters.^7^ The use of routine data to measure and improve quality of primary healthcare delivery, while common in high-income countries,^32^ is rare in LMICs.^20^ The Guidelines Adherence in Slums Project has pioneered the use of rubber stamp templates and smartphones to deliver routine data on primary care delivery in low-resource settings. A previous publication described improvements in clinical documentation for non-communicable diseases.^23^ This paper first demonstrates improved documentation of care for common infectious conditions. It then provides insights gained from improved documentation (extent of antibiotic use in managing common infections in private sector PHCs serving the informal settlements of Nairobi), offers possible reasons for antibiotic overuse and highlights differences in clinical practice resulting from the intervention.

### Antibiotic use in primary care

Antibiotic prescription rates for any of the four infectious diseases in this study, across different PHCs, were over 90%. Broad-spectrum antibiotics were frequently prescribed. Amoxicillin or amoxicillin/clavulanate (41%–70%) and cephalosporins (21%–53%) together accounted for about 90% of antibiotic prescriptions for URTI; metronidazole was used in 53%–97% of all GI encounters in children (table 2). Surveillance studies in South Africa and India using prescription and sales records reveal antibiotic prescription rates between 21% and 43% of all patients seen/prescriptions dispensed.^33 34^ Like in Kenya, broad-spectrum antibiotics were frequently prescribed in South Africa and India. But unlike in India, where the most common antibiotics used for gynaecological symptoms were doxycycline, ciprofloxacin and trimethoprim/sulfamethoxazole,^34^ antibiotics commonly prescribed for STI in this study were macrolides, metronidazole and benzathine penicillin G. Variations in antibiotic use are, however, likely across different settings within countries,^7 33 34^ which was not explored in this study.

While not directly comparable to the data from studies using prescriptions/sales data,^7^ routine data on case-specific use of antibiotics highlight the extent of antibiotic use in LMICs in comparison to similar data from high-income countries.^35 36^ Where available, case-specific prescribing data in LMICs reveal similarly high rates of antibiotic use.^37^ Since ‘ideal’ prescribing rates are likely to vary by infectious condition,^35 36^ surveillance using prescription or sales data alone (without information on diagnosis or documentation on the need for antibiotics) may hide differences in case mix and context of care. Routine data on primary care delivery, combined with audit and feedback, also has the advantage of being actionable at facility level,^37 38^ and presents a powerful and widely studied and used tool to affect clinician practices.^13^ This study argues for a similar approach to combat global AMR in LMICs.

### Knowledge gaps and know-do gaps

Routine data concurrently provide detailed information on case management, with inferences possible on the appropriateness of antibiotic use. In PHCs where UTI charts were audited, UTIs were frequently codiagnosed and managed with STIs. While distinguishing between the two can be challenging^39^ and one is often missed when diagnosing and treating the other,^39 40^ chart audits revealed that antibiotics for STIs were prescribed in more than half of all UTI cases, often with no documentation justifying such use. Qualitative data from the Guidelines Adherence in Slums Project also indicate that non-physician clinicians often treat the two conditions interchangeably (manuscript in preparation). This gap in clinician knowledge likely contributed to the high number of antibiotics prescribed in UTI cases (1.79±0.09 antibiotics per encounter) even in the postintervention period.

Appropriateness and/or availability of nitrofurantoin to manage uncomplicated cystitis^25 29^ was another ‘knowledge gap’ identified from chart audits and supported by interviews with non-physician clinicians (manuscript in preparation). A shift from the use of broad-spectrum antibiotics (quinolones, amoxicillin) to narrow-spectrum antibiotics (nitrofurantoin) for uncomplicated cystitis was seen following the intervention, which included CME sessions on relevant guidelines and feedback of PHC-specific practice data. This suggests that knowledge gaps, once identified, could be closed through targeted clinician training initiatives where feedback of prescribing data along with specific guideline recommendations could nudge provider behaviour towards more rational antimicrobial use.^41^ Such monitoring of clinician knowledge, and targeted training, could be included into the core elements of global antimicrobial stewardship.^42^

Guidelines often help identify situations warranting antibiotic use—for example, the modified McIsaac/Centor criteria to identify tonsillitis caused by group A streptococcae.^43^ Templates for URTI and GI contained documentation fields for when criteria for antibiotic prescription were met (figure 1), and the data reveal a know-do gap between clinicians’ knowledge of what constitutes appropriate care (recognising situations where antibiotic use is not justified) and the actual care delivered (use of antibiotics regardless).^10 44^ Non-physician clinicians in LMICs are, therefore, likely aware of the need to reduce unnecessary antibiotic prescriptions, but pressures like meeting patient and management expectations (unpublished qualitative data) may continue to drive inappropriate antibiotic use despite knowledge of evidence-based recommendations. Clinician training alone might not be sufficient to overcome know-do gaps, and better monitoring and incentives would be needed for clinicians to deliver the care they know is appropriate.^44^

### Strengths and limitations of the study

The study aimed to test the use and acceptance of a novel approach to improve clinical documentation. Besides the small scale of the study, baseline data were often not available for audit because records are frequently not maintained at facilities. Across the project, data from templates were received from seven of nine PHCs, but charts were available for audit from only four. The remaining did not maintain patient records in the facility, highlighting a major challenge to access patient-level primary care data in LMICs. Future studies should account for variations in workflows, capacities and priorities among different facilities. While this study defined clinical documentation measures that could be compared before and after the intervention, it did not predefine outcomes in clinical quality. Future studies could also focus on measuring quality indicators and outcomes in a randomised, controlled design.^45^ The study also did not aim to evaluate the impact of CMEs (or other elements of the intervention) independent of template use.

Templates can and are easily modified to suit the workflows of each PHC (or QI initiative). The approach is, therefore, well-suited to continuous QI cycles, where templates can be modified based on targets achieved without burdening clinicians with documentation tasks. Modifications to templates could be made to improve, for example, differentiation between UTIs and STIs, and once this is achieved further changes could address other gaps in quality. Such a stepwise approach that customises interventions to the performance at facility level has been shown to be effective in other QI initiatives in similar settings.^14^ Lessons on improvements to template design include the use of specific drug names like ciprofloxacin and norfloxacin instead of less familiar antibiotic class names like quinolones (a possible reason for poor concordance of quinolone use between charts and templates).

### Role of mobile technology in primary healthcare

Mobile technologies can improve primary healthcare in diverse ways,^46^ but there is growing recognition that the focus on technology often ignores other factors critical to the success of digital health interventions.^19 22^ The ‘appropriate technology’ of rubber stamp templates addresses LMIC constraints (eg, financial and non-financial costs of training non-physician clinicians on computer-based information systems)^47^ and takes advantage of the ubiquitous availability and use of both paper and smartphones. While all data in this study were manually extracted from template images taken and shared using mobile phones, a computer vision-based smartphone app to automatically extract information entered in paper templates into digital data has been field tested and deployed. The findings reported in this study make the case for the usefulness and appropriateness of the hybrid, paper-to-digital approach to implement routine data collection. Collection of routine data through such a hybrid m-Health system could enable both QI initiatives (through regular audit and feedback cycles) as well as CDS systems, which rely on more immediate feedback that mobile technology enables.^48^

## Conclusion

AMR is one of the major global public health challenges and requires concerted stewardship efforts at local, national and international levels to avert rising morbidity and mortality from untreatable infections.^1 2 42^ While overprescribing of antibiotics in primary care contributes directly to AMR,^3 35^ its extent is surprisingly poorly understood and monitored in LMIC settings.^7^ This study used routine data to reveal the extent of antibiotic overuse in the treatment of commonly encountered infectious conditions in PHCs in LMICs—over 90% of patient encounters resulting in antibiotic prescription, with more than one antibiotic prescribed on average per encounter. Lack of clinician knowledge contributes to inappropriate prescription and can be overcome through feedback and training, but know-do gaps also contribute and likely require interventions at individual (client and clinician), institutional and system levels to overcome.^2 49^ PHCs in LMICs, and the non-physician clinicians that staff them, are at the frontier of the battle against AMR, and the sustainable generation of routine case management data from these facilities is critical to effective antimicrobial stewardship. An unlikely partnership between the ancient technology of rubber stamps and modern mobile phones may hold the key.
